# Human Spontaneous Combustion

**Published:** 1889-11

**Authors:** 


### HUMAN SPONTANEOUS COMBUSTION.

Twelve years ago last August, a man met death in a peculiar and horrible manner in San Francisco. James Harley, the victim, had just recovered from an attack of delirium tremens. He had been a regular sot and was thoroughly soaked with alcohol. His last attack of the "jim jams" was the third he had undergone within a few months. On the morning in question he had started on another spree. At about 11 o'clock in the day, he had about all the liquor on board he was able to carry. Stepping into a saloon on one of the principal streets he called for a swig of "bug juice," which was promptly refused, the bartender noting the condition of the man and remembering his late battle with the snakes. Harley scowled and turned to a gas jet to light his pipe. A second later there was a drunken moan, a flash of alcoholic flame, and Harley fell heavily to the floor, his head and neck veiled in smoke, while blue jets of flame were issuing from his ears, mouth and nostrils. As soon as water could be procured (water was a commodity rather scarce around such places) it was dashed into the sufferer's face, not in time, however to save his life. The face was as black is that of a negro. The ears were charred, the inside of the mouth black and the tongue burned to a crisp.

